# Anatomo-sonographic identification of the longissimus capitis and splenius cervicis muscles: principles for possible application to ultrasound-guided botulinum toxin injections in cervical dystonia

**DOI:** 10.1007/s00276-020-02646-w

**Published:** 2021-01-18

**Authors:** Eleonore Brumpt, Sebastien Aubry, Fabrice Vuillier, Laurent Tatu

**Affiliations:** 1 Department of Radiology, Centre Hospitalier Universitaire de Besançon, University of Franche-Comté, 3 Boulevard Fleming, 25000 Besançon, France; 2 Department of Anatomy, University of Franche-Comté, 25000 Besançon, France; 3 Department of Neuromuscular Diseases, Centre Hospitalier Universitaire de Besançon, Boulevard Fleming, 25000 Besançon, France; 4 Department of Neurology, Centre Hospitalier Universitaire de Besançon, Boulevard Fleming, 25000 Besançon, France; 5 Nanomedecine Laboratory, INSERM EA4662, University of Franche-Comte, 25000 Besançon, France

**Keywords:** Botulinum toxin, Cervical dystonia, Ultrasound, Anatomic landmarks, Neck muscles

## Abstract

**Objective:**

The main objective of this study was to define and verify anatomo-sonographic landmarks for ultrasound-guided injection of botulinum toxin into the longissimus capitis (LC) and splenius cervicis (SC) muscles.

**Methods and results:**

After a preliminary work of anatomical description of the LC and SC muscles, we identified these muscles on two cadavers and then on a healthy volunteer using ultrasound and magnetic resonance imaging (MRI) to establish a radio-anatomical correlation. We defined an anatomo-sonographic landmark for the injection of each of these muscles. The correct positioning of vascular glue into the LC muscle and a metal clip into the SC muscle of a fresh cadaver as verified by dissection confirmed the utility of the selected landmarks.

**Discussion:**

For the LC muscle, the intramuscular tendon of the cranial part of the muscle appears to be a reliable anatomical landmark. The ultrasound-guided injection can be performed within the cranial portion of the muscle, between the intra-muscular tendon and insertion into the mastoid process at dens of the axis level. For the SC muscle, the surface topographic landmarks of the spinous processes of the C4–C5 vertebrae and the muscle body of the levator scapulae muscle seem to be reliable landmarks. From these, the ultrasound-guided injection can be carried out laterally by transfixing the body of the levator scapulae.

**Conclusion:**

The study defined two cervical anatomo-sonographic landmarks for injecting the LC and SC muscles.

## Introduction

The posterior neck muscles are the major actors in cervical dystonia. Botulinum toxin treatment is the gold standard in the treatment of these dystonias. However, the treatment can fail for multiple reasons [2], including poor targeting of the muscles to be treated. Anatomical knowledge of the cervical muscles is, therefore, essential to treat them by botulinum toxin injection [9]. Some muscles are identified using surface anatomy and then injected under electromyographic (EMG) guidance. EMG guidance provides the clinician with information about the electrical activity of the muscle and the type of involvement of each muscle (dystonia, tremors, compensation, etc.). It allows practitioners to avoid injecting non-muscular structures or muscles that are not involved [6]. Nevertheless, some muscles remain difficult to identify. The lateral region of the posterior cervical muscles is one such area, and three muscles that are important in the treatment of cervical dystonia are closely linked to it. These are the splenius capitis (SC), longissimus capitis (LC), and longissimus cervicis muscles. Their individualization is random, especially at the C4–C5 spinal level, where they form a common lateral muscle mass. This justifies the use of imaging in combination with EMG. Ultrasound is the most suitable imaging method for this detection because of its ease of access, maneuverability, excellent spatial resolution, absence of irradiation, and low cost. However, there is currently no anatomo-sonographic landmark for SC and LC muscles. Our initial hypothesis was to inject the cranial part of the LC muscle by transfixing the sternocleidomastoid and splenius capitis muscles in an ultrasound-guided procedure and to inject the SC muscle by transfixing the levator scapulae muscle in its portion adjacent to the splenius capitis muscle.

The objective of this work was to define and verify anatomo-sonographic landmarks for selective injection of botulinum toxin into the LC and SC muscles using a both ultrasound and EMG guidance.

## Materials and methods

The study was carried out at the University of Franche-Comté, within the anatomy laboratory and the EA 481 (Neurosciences) and EA 4262 (Nanomedicine, Imaging and Therapeutics) groups. The work was carried out in two stages. The first step was to define the normal anatomy of the SC and LC muscles and to select anatomo-sonographic landmarks to target them for injection. The second step was to validate the selected anatomo-sonographic landmarks.

We studied the normal anatomy of the SC and LC muscles by dissecting two male fixed cadavers embalmed using the Winckler method (age at death 65 and 68 years) and by analyzing virtual anatomical sections using the Anatomage digital anatomy table. Ultrasound anatomy was defined by the exploration of a healthy 24-year-old female volunteer subject using an Aixplorer ultrasound scanner (Supersonic Imagine, Aix-en-Provence, France) equipped with a high-frequency linear probe (5–18 MHz). On the same healthy subject, the correspondence with the cross-sectional imaging was performed using 3-T magnetic resonance imaging (MRI) in T1-weighted anatomical sequences (*Skyra 3 T, Siemens, Erlangen, Germany*). The comparison of these two imaging examinations enabled us to choose the anatomo-sonographic landmarks for the SC and LC muscles.

The anatomo-sonographic landmarks were validated by a study on a fresh cadaver (age at death 70 years). A 3-Tesla MRI (*Skyra 3 T, Siemens, Erlangen, Germany*) of the cervical region was performed in T1-weighted anatomical sequences to ensure that there was no pathological condition or anatomical variant of the posterolateral cervical region. Under the normal conditions for botulinum toxin injection, and using the defined anatomo-sonographic landmarks, we implanted a metal clip into the SC muscle and injected 1 mL of surgical glue (Glubran) into the LC muscle, under ultrasound guidance (Esaote ultrasound scanner, Kontron Medical with high-frequency linear probe from 3 to 13 MHz). We then performed a targeted anatomical dissection to verify the positioning of the clip and glue.

## Results

### Normal morphological anatomy of the SC and LC muscles

**Fig. 1:**
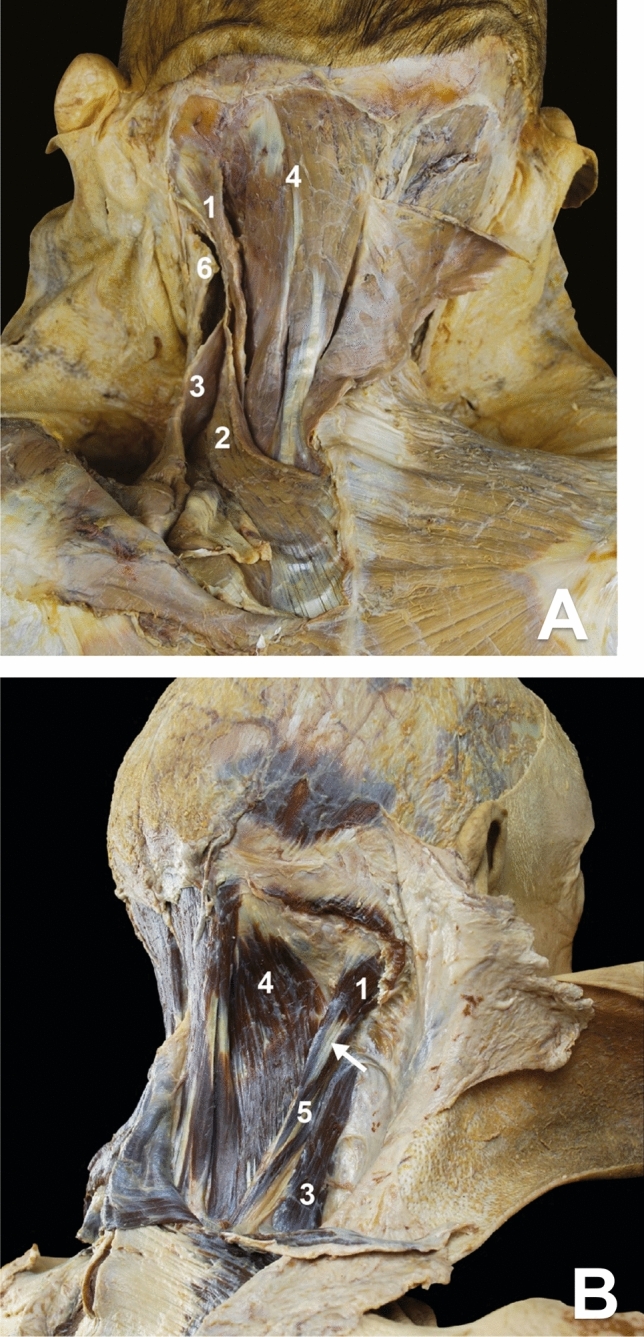
**a** Dissection of the posterior cervical muscles (left side, fixed cadaver). Longissimus capitis (1), splenius cervicis (2), levator scapulae (3) semi-spinalis capitis (4), and sternocleidomastoid muscle (6). **b** Dissection of the posterior cervical muscles (right side, fixed cadaver). Longissimus capitis (1) with its intra-muscular tendon (arrow), levator scapulae (3), semi-spinalis capitis (4), longissimus cervicis (5). Splenius capitis has been cut

**Fig. 2:**
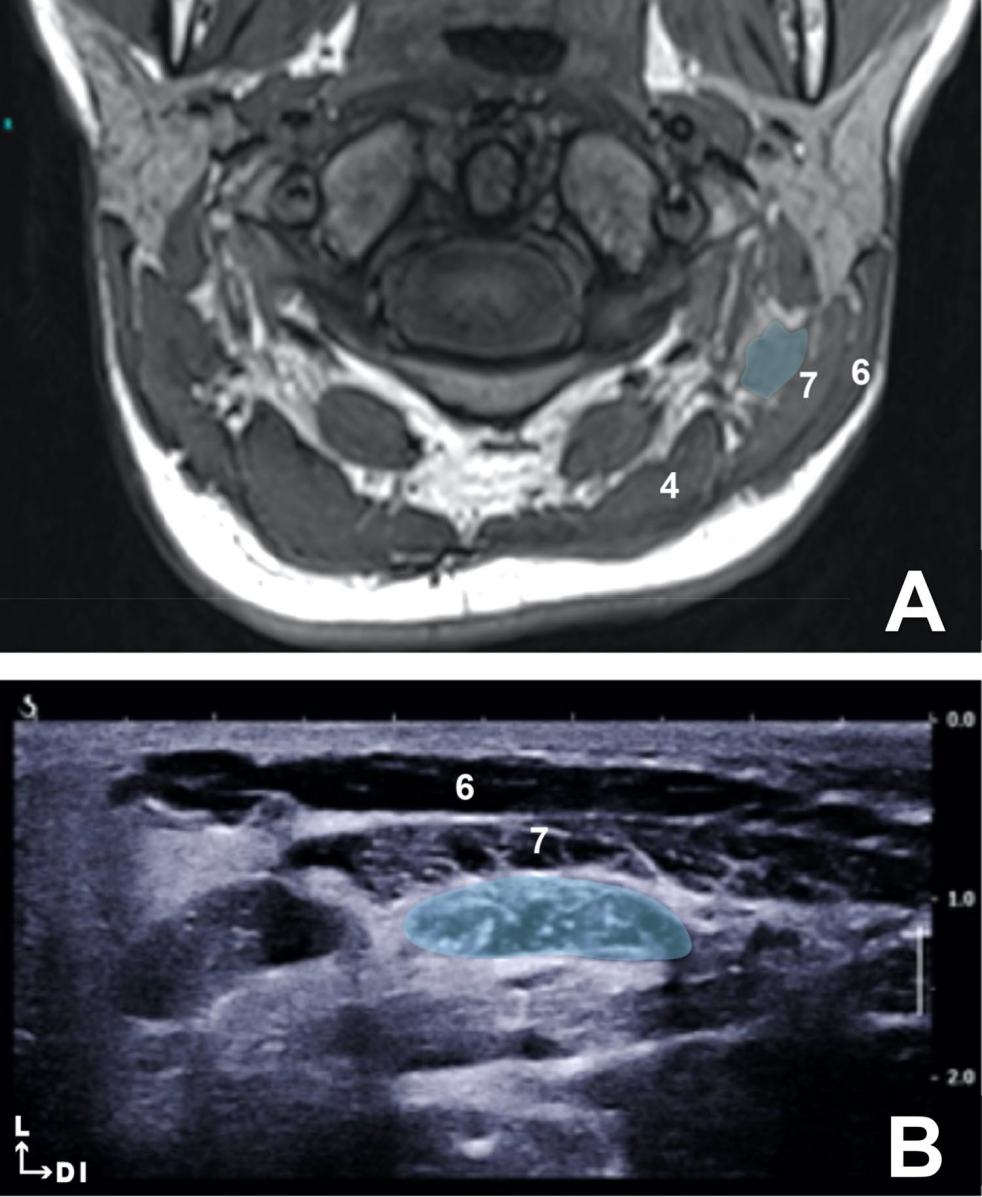
**a** T1-weighted MRI, axial slice at level of the dens of axis. Longissimus capitis (blue). Sternocleidomastoid muscle (6), splenius capitis (7), and semi-spinalis capitis (4). **b** Ultrasound image at the level of the dens of axis. Longissimus capitis (blue). Sternocleidomastoid muscle (6) and splenius capitis (7)

We studied the anatomy by dissecting two fixed cadavers and using the Anatomage anatomy table. The LC muscle originated from the transverse processes of the C3–T3 vertebrae and inserted into the mastoid process. We observed a small intramuscular tendon close to its insertion on the mastoid process. The orientation of the muscle was oblique upwards, forwards, and outwards. The LC muscle, initially located in a deep plane, gradually became superficial compared to the longissimus cervicis, the SC and the levator scapulae muscles. It remained deep to the splenius capitis muscle. On the mastoid process, the insertions of the sternocleidomastoid muscle covered the LC and SC muscles (Figs. 1 and 2).

The SC muscle originated from the spinous processes of the T3–T5 vertebrae and inserted on the transverse processes of the C1 and C2 vertebrae. At the C4–C5 level, the SC muscle was the most superficial muscle of the common lateral mass. It was immediately deep to the levator scapulae muscle. Its orientation was oblique, upwards, forwards and outwards (Figs. 1 and 2).

### Choice of anatomo-sonographic landmarks

Ultrasound anatomy was studied in a healthy volunteer subject, in correlation with MRI images performed for the same volunteer. We proposed an injection site and anatomo-sonographic landmarks for the two muscles studied.

#### LC muscle

**Fig. 3:**
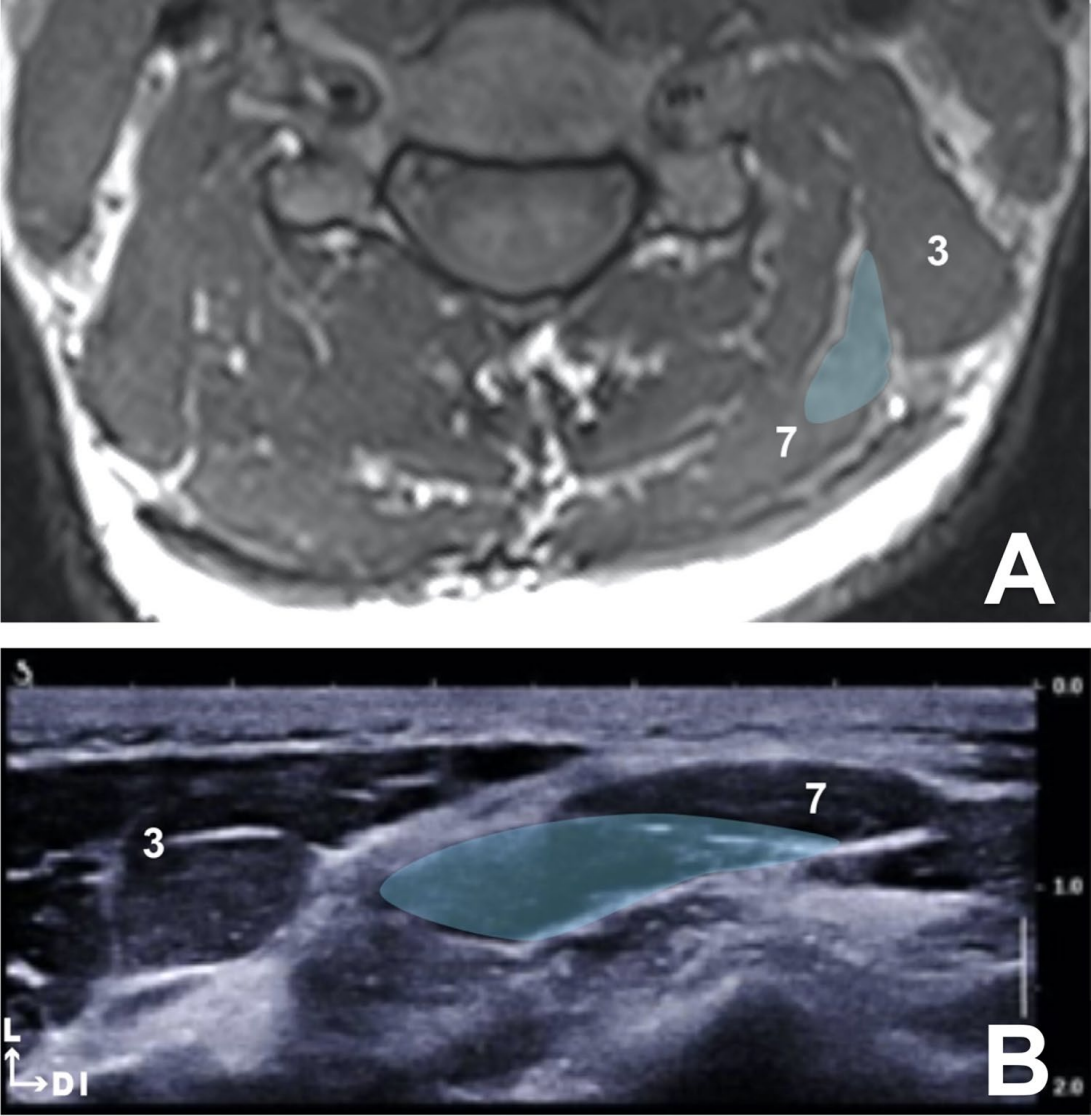
**a** T1-weighted MRI axial slice at the C5 vertebrae level. Splenius cervicis (blue). Levator scapulae (3) and splenius capitis (7). **b** Ultrasound image between C4 and C5 spinous processes. Splenius cervicis (blue). Levator scapulae (3) and splenius capitis (7)

The intramuscular tendon identified during dissection of the fixed cadaver was visualized using ultrasound and MRI images. It was located in the most cranial portion of the muscle between the C2 vertebra and the mastoid process. We chose to use this intramuscular tendon as a topographic reference for the injection of the LC muscle. The proposed site for the injection of this muscle was between the mastoid process and this intramuscular tendon, i.e. at the level of the C2 vertebra (Fig. 3).

#### SC muscle

**Fig. 4:**
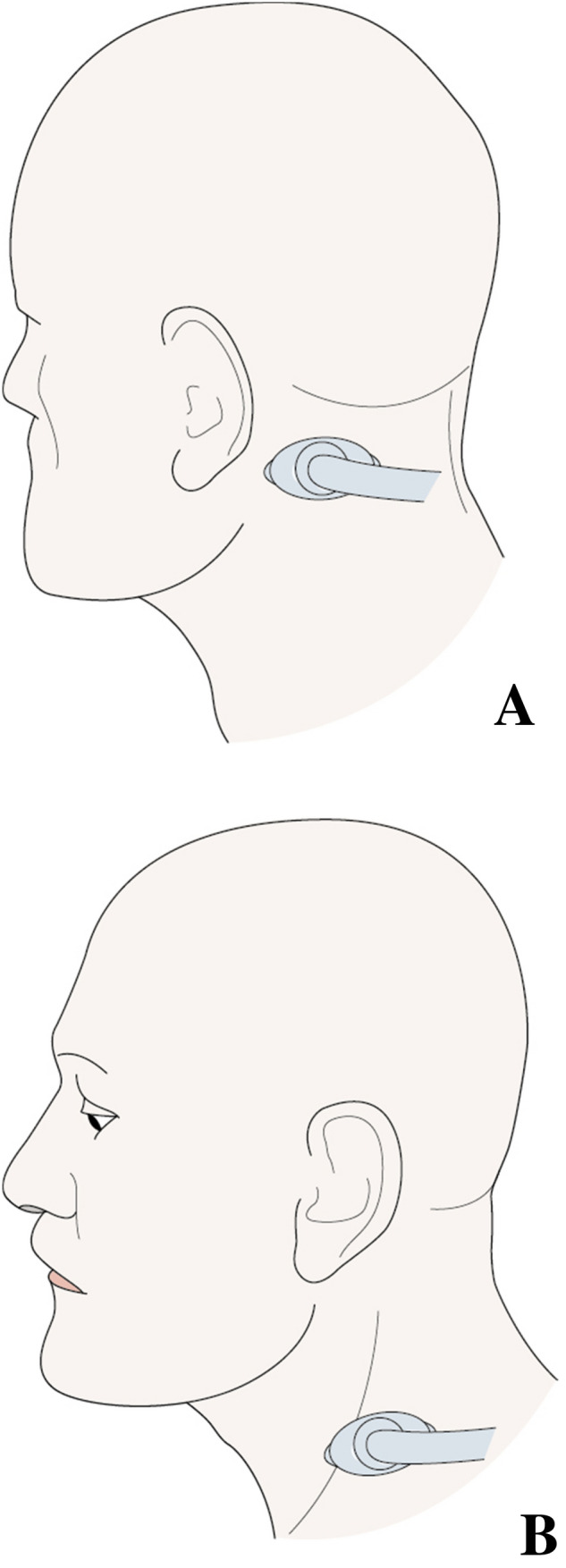
**a** Probe position for longissimus capitis injection. **b** Probe position for splenius cervicis injection

At the level of the C4–C5 vertebrae, the SC muscle was located deep to the levator scapulae muscle. The dynamic lateral ultrasound scanning of its downward, backward and inward orientation enabled us to locate it with certainty. For the injection of this muscle, we chose to use the topographic surface landmarks of the spinous processes of the C4–C5 vertebrae and the muscle body of the levator scapulae muscle in the posterior triangle of the neck (Fig. 4).

### Validation of the selected anatomo-sonographic landmarks

The validation of the landmarks was performed on a fresh cadaver under the conditions of botulinum toxin injection. Prior to this validation, we performed a T1-weighted sequence MRI of the cadaver to verify that there was no anatomical variant in the cervical region.

#### LC muscle

**Fig. 5:**
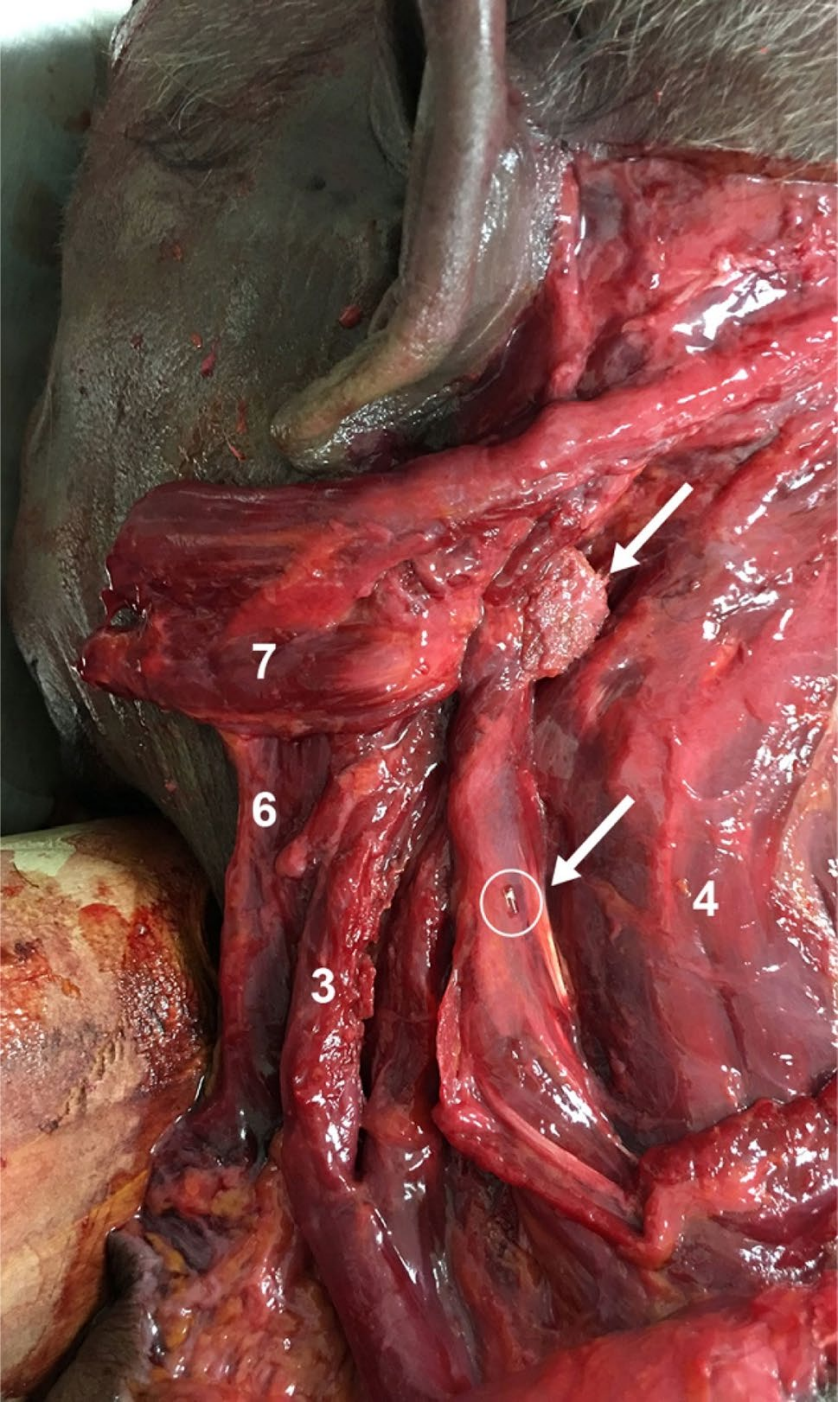
Dissection of the posterior cervical muscles (left side, fresh frozen cadaver). Metal clip (circle and arrow) within the splenius cervicis. Solidified surgical glue (arrow) within the longissimus capitis (6), levator scapulae (3), semi-spinalis capitis (4), sternocleidomastoid muscle (6), and splenius capitis (7)

Using the chosen landmark, namely the intramuscular tendon at the C2 vertebrae, 1 mL of Glubran surgical glue was injected under ultrasound guidance. Dissection of the sub-occipital region of the fresh cadaver confirmed the correct positioning of the surgical glue within the LC muscle (Fig. 5).

#### SC muscle

Using the selected landmarks, namely the spinous processes of the C4–C5 vertebrae and the muscular body of the levator scapulae, a metal clip was inserted into the SC muscle under ultrasound guidance. Dissection of the lateral cervical region of the cadaver confirmed the correct positioning of the clip within the SC muscle (Fig. 5).

## Discussion

The defined and validated anatomo-sonographic landmarks allow the injection of the LC muscle in its cranial portion by transfixing the sternocleidomastoid and splenius capitis muscles, and the injection of the SC muscle in its portion adjacent to the splenius capitis muscle by transfixing the levator scapulae muscle.

Cervical dystonia is the most common focal dystonia. The standard treatment is targeted injections of botulinum toxin, the prerequisite for which is a relevant semiological analysis using the *collum-caput concept* [7] and a knowledge of cervical muscle anatomy [9]. The choice of muscles to inject is also based on semiological analysis [3]. It is, therefore, essential to be able to target the chosen muscles as effectively as possible. The injections are carried out under electromyographic guidance, which enables the identification of the dystonic muscles and avoidance of the muscles that are not involved or are compensating. The introduction of the ultrasound tool in the treatment of cervical dystonia is recent [8] and should allow better targeting of muscles that are difficult to inject. The common lateral muscle mass of the middle layer of the posterior cervical muscles—which includes the LC, SC and longissimus cervicis muscles—is one these difficult areas. Ideally, a combination of electromyography and ultrasound would be used.

The frequency of the presence of the intramuscular tendon at the cranial part of the LC muscle in the population is not specified in the anatomical literature. However, the presence of this tendon has already been observed and reported [4]. It would be interesting to analyze this frequency by ultrasound and/or MRI study of the cervical region. This tendon is a good reference for the injection of the LC muscle but requires the use of ultrasound, as does the injection of the SC muscle through the levator scapulae muscle.

It is possible to propose an injection methodology for the two muscles studied. For the injection of the LC muscle, it is first necessary to place the ultrasound probe on the mastoid process, then move it down and back following the anatomical orientation of the LC muscle. The intramuscular tendon is then visible at the level of the C2 vertebrae. The injection is performed within the cranial portion of the muscle, between the intramuscular tendon and insertion into the mastoid process. In the absence of the intramuscular tendon, it is recommended not to cross the horizontal line passing through the C2 spinous process, below which the LC muscle becomes more difficult to visualize because of its deep location. At this level, it already belongs to the common lateral muscle mass with the longissimus cervicis and SC muscles.

For the injection of the SC muscle, it is necessary to use surface topographic landmarks, consisting of the spinous processes of the C4 and C5 vertebrae and the muscle body of the levator scapulae muscle in the posterior triangle of the neck. The injection is carried out anteroposteriorly, passing through the levator scapulae muscle in a space located in the craniocaudal plane between the horizontal lines that pass through the spinous processes of the C4 and C5 vertebrae.

The choice of injection site at the upper part of the LC does not correspond to the usual maximum concentration area of neuromuscular junctions within a muscle. It is generally accepted that this area is equidistant from distal and proximal muscle insertion tendons. However, this statement has not been validated for all cervical muscles. For example, the distribution of neuromuscular junction areas within the sternocleidomastoid and splenius capitis muscles does not follow this rule [1, 5].

Our study only included a small number of subjects, all of whom had an intramuscular tendon at the cranial part of the LC muscle. The definition of this tendon frequency among the population would reinforce the external validity of our study. Establishing the clinical relevance of these landmarks would require a prospective comparative study of the results of LC and SC muscle botulinum toxin injections under ultrasound guidance in combination with electromyography, compared to the results of injections under electromyographic guidance alone.

The third component of the common lateral muscle mass is the longissimus cervicis muscle. It seemed difficult to us to propose reliable anatomo-sonographic landmarks for the injection of this muscle. Indeed, its morphological characteristics (thinness and entanglement with the surrounding muscles) still limit its detection in ultrasound and MRI.

## Conclusion

This study defined reliable anatomo-sonographic landmarks that are useful for the injection of botulinum toxin into the LC and SC muscles. They enable a better ultrasound-guided injection of botulinum toxin and should help to improve therapeutic effectiveness in cervical dystonia.

## Abbreviations

EMG: Electromyographic
LC: Longissimus capitis
MRI: Magnetic resonance imaging
SC: Splenius cervicis
